# Toxic Epidermal Necrolysis-Like Acute Cutaneous Lupus Erythematosus With Histiocytic Necrotizing Lymphadenitis: A Case Report

**DOI:** 10.7759/cureus.79275

**Published:** 2025-02-19

**Authors:** Adam Cardenas, Edgar Martinez, Khang Nguyen

**Affiliations:** 1 Dermatology, Dell Medical School, University of Texas at Austin, Austin, USA

**Keywords:** acute cutaneous lupus erythematosus, derm path, derm-rheum, systemic lupus erythematosus, toxic epidermal necrolysis (ten)

## Abstract

Toxic epidermal necrolysis-like acute cutaneous lupus erythematosus (TEN-like ACLE) is an acute, life-threatening manifestation of ACLE. Histiocytic necrotizing lymphadenitis (HNL) is a rare disease of lymph nodes that presents with fever and lymphadenopathy and is generally self-resolving.

We report a case of a 48-year-old female who presented to the emergency department (ED) with fever and arthralgias in the setting of 30 lbs weight loss over two months. She had self-discharged and re-presented to the ED three weeks later with widespread, painful skin sloughing. On exam, the patient had scattered, violaceous macules coalescing into patches, flaccid bullae, and erosions on her scalp, face, upper central chest, shoulders, arms, and lower legs. She had no oral, ocular, or genital mucosal involvement. Biopsy showed vacuolar interface dermatitis with granular deposits of IgM, IgG, and C3 along the basement membrane zone. A diagnosis of TEN-like ACLE complicated by HNL was made, given the clinical picture and biopsy findings. The patient showed improvement with topical triamcinolone and oral corticosteroid but ultimately passed away secondary to a spontaneous retroperitoneal bleed.

This case highlights the clinical features and management of TEN-like ACLE, as well as its correlation with HNL, to facilitate earlier detection and intervention.

## Introduction

Systemic lupus erythematosus (SLE) is a multiorgan, autoimmune condition of unknown etiology. Signs and symptoms include fatigue, weight loss, fever, arthralgias, rash, and myalgia. Autoantibodies play a pivotal role in diagnosis, with anti-dsDNA and anti-Smith antibodies being specific markers for SLE [1].

Toxic epidermal necrolysis (TEN)-like eruptions are clinical presentations similar to drug-induced TEN, characterized by painful, acute skin blistering and denudation and positive Nikolsky sign. However, while classic TEN is drug-induced, TEN-like eruptions have been reported to occur with underlying autoimmune conditions, graft-versus-host disease (GVHD), and pseudoporphyria. Both drug-induced TEN and TEN-like eruptions have similar histopathological changes characterized by acute and massive cleavage of the epidermis resulting from hyperacute epidermal basal cell apoptotic injury. It takes a high index of suspicion, recognition of patterns on physical exam, and appropriate laboratory investigation for a clinician to distinguish drug-induced classic TEN from TEN-like eruptions [2,3].

Histiocytic necrotizing lymphadenitis (HNL) (also known as Kikuchi-Fujimoto disease) is a rare lymphohistiocytic disorder of unknown etiology characterized by painful lymphadenopathy and systemic symptoms including fever, malaise, and arthralgias. It is diagnosed through lymph node biopsy, which typically reveals paracortical foci with histiocytic infiltrate. HNL is typically benign and self-resolving, yet it is frequently initially misdiagnosed as a sign of infection or malignancy. In rare cases, HNL can be associated with SLE and can present before, simultaneously, or after the diagnosis of SLE has been established. While SLE is the most commonly associated autoimmune condition with HNL, there are fewer than 20 reported cases of HNL occurring in the setting of SLE in the literature [4,5].

We present a unique case of a patient who was initially diagnosed with HNL, which progressed to TEN-like acute cutaneous lupus erythematosus (ACLE).

## Case presentation

A 48-year-old woman presented to the emergency department (ED) with a two-week history of unexplained persistent fever and arthralgias and a two-month history of unexplained 20-pound weight loss. Laboratory workup is shown in Table 1. Abdominal computed tomography showed multiple splenic infarcts along with retroperitoneal and left inguinal lymphadenopathy. A periaortic lymph node biopsy revealed lymphohistiocytic necrotizing lymphadenitis, consistent with HNL. Simultaneous laboratory workups and reviews of symptoms raised concerns about HNL in the SLE setting. Unfortunately, the patient self-discharged before her workup was complete.

**Table 1:** Pertinent lab values at initial visit to the emergency department.

Lab	Value	Reference range
Antinuclear antibody	1:2560, homogenous pattern	Varies
Anti-chromatin antibody	>8.0 AI	Negative: <1.0 AI
Anti-double-stranded DNA antibody	12 IU/mL	Negative: <4.9 IU/mL
C-reactive protein	16.6 mg/dL	0.0-0.08 mg/dL
C3	56 mg/dL	88-201 mg/dL
C4	26 mg/dL	15-45 mg/dL

**Figure 1:**
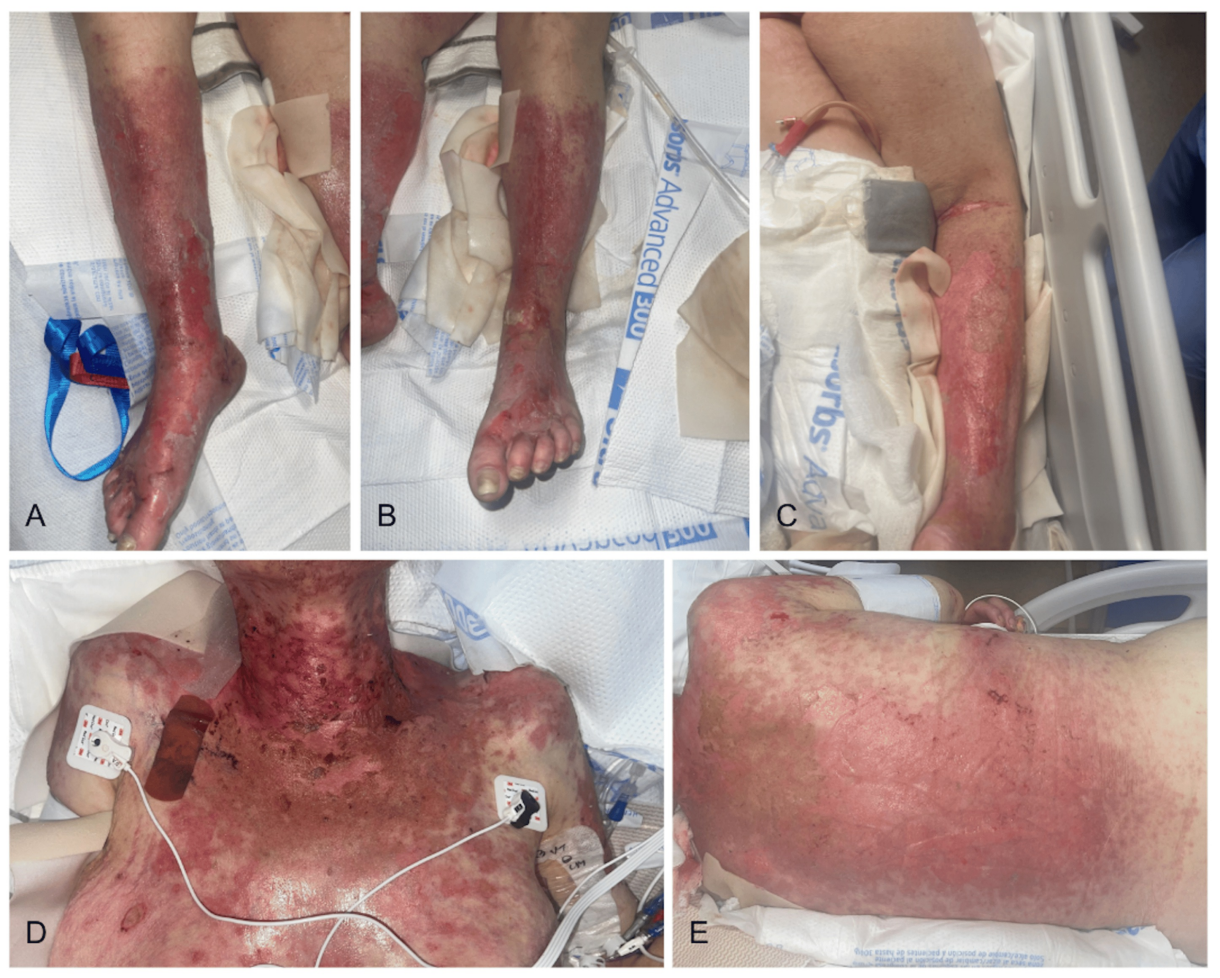
Physical presentation at the emergency department concerning for toxic epidermal necrolysis. (A) Right lower leg with skin sloughing and denudation on erythematosus background. (B) Left leg blistering with sloughing of skin. (C) Left forearm skin denudation. (D) Upper chest with scattered erosions and hemorrhagic crusting. (E) Back with the widespread skin denudation.

Three weeks later, the patient was re-presented to the ED with a one-week history of an intensely painful, widespread blistering rash. Physical examination is shown in Figure 1. Notably, there was a sharp line of demarcation sparing the superior shins, thighs, flexor surfaces, and other photoprotected areas. There was no intra-oral, ocular, or genital mucosal involvement.

**Figure 2:**
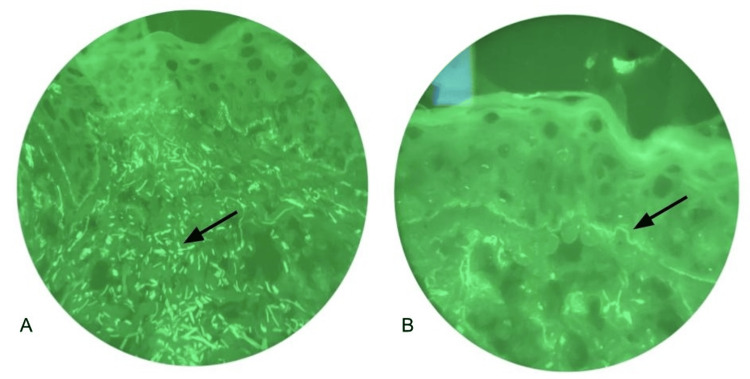
Direct immunofluorescence (DIF) staining performed on a lesional punch biopsy from the shoulder region. (A) DIF highlighting C3 deposition along the basement membrane. (B) IgM linear deposition along the basement membrane.

Hematoxylin and eosin (H&E) staining on lesional and perilesional biopsies revealed vacuolar interface dermatitis with focal epidermal ulceration and superficial-to-mid perivascular dermatitis of predominantly lymphoid origin. Direct immunofluorescence (DIF) is shown in Figure 2. Based on these factors, the diagnosis of TEN-like ACLE was made.

The patient was started on wet wraps with triamcinolone 0.1% ointment, oral prednisone 1 mg/kg daily, pain control with intravenous and oral narcotics, and heparin drip for splenic infarcts. She showed noticeable improvements within days, with skin re-epithelialization and fading erythema. Unfortunately, the patient passed away nine days after admission, secondary to a sudden retroperitoneal bleed, possibly in the setting of a recent retroperitoneal lymph node biopsy and anticoagulation for splenic infarct.

## Discussion

We present a rare case of TEN-like ACLE eruption, which was complicated by HNL in the setting of SLE. Our patient’s case is unique in that her acute cutaneous lupus flare presented very much like classic drug-induced TEN, which required recognition of photo-distributed patterns and careful medication history reconciliation to distinguish between the two. To the best of our knowledge, less than 45 cases of TEN-like ACLE eruptions have been reported as of 2021 [6,7]. There are even fewer reported cases of concomitant HNL in the setting of SLE in the same patient.

TEN-like eruptions fall under the umbrella of the acute syndrome of apoptotic panepidermolysis (ASAP), which includes classic drug-induced TEN, TEN-like SLE, TEN-like GVHD, and TEN-like pseudoporphyria. The clinical presentations of these eruptions have immense overlap, with the leading symptoms being acute, painful skin blistering, and denudation. However, specifically for TEN-like ACLE eruption, the rash is localized and more intense in sun-exposed regions [7,8].

Delays in diagnosis and treatment are not infrequent. TEN-Like lupus also can be misdiagnosed as drug-induced TEN/Stevens-Johnson syndrome (SJS), erythema multiforme, bullous systemic lupus, dermatomyositis, pemphigus vulgaris, paraneoplastic pemphigus, bullous pemphigoid, and Rowell syndrome, among others. For these reasons, a detailed physical examination and accurate history collection, with emphasis on possible triggers, is needed for rapid recognition of this condition. A biopsy can aid in the diagnosis, which will show full-thickness epidermal necrosis with sparse lymphocytic infiltrate and granular deposition of IgM, IgG, and C3 along the basement membrane zone on histology and DIF studies of the skin, respectively [7-9].

Management of TEN-like ACLE includes either a topical corticosteroid or topical calcineurin inhibitor paired with hydroxychloroquine or chloroquine [10]. For the acute exacerbation seen in these cases, it is also important to consider systemic corticosteroids. Depending on the presence of bullous lesions, dapsone or methotrexate could be explored as second-line agents if no improvement is seen.

It is important to note the prognosis for the diagnoses differ drastically. For TEN, the mortality risk is 25-35% [3]. On the other hand, patients with TEN-like ACLE have a lower 11% mortality rate [11]. The range of outcomes further highlights the need to carefully evaluate these patients comprehensively, integrating the clinical presentation, past medical history, biopsy findings, and possible exposures. 

For our specific case, the patient was diagnosed with HNL, which has been brought forth by case studies to possibly be associated with SLE [11,12]. However, few sources describe such an acute presentation which necessitates rapid intervention. Through recognition of the association between the two conditions, one could potentially engage with diagnostic or therapeutic management earlier.

## Conclusions

Our patient’s case is unique in that her acute cutaneous lupus flare presented very much like an SJS/TEN eruption, which required recognition of a photo-distributed pattern and careful medication history reconciliation to distinguish between the two. Becoming familiar with the myriad of clinical manifestations of ACLE and TEN-like eruptions is crucial in preventing delay in diagnosis. In addition, identifying the symptoms and radiographic findings of HNL and its correlation to SLE can help prevent delays in treatment.
